# Identification of a novel mutation in the *ABCA4* gene in a Chinese family with retinitis pigmentosa using exome sequencing

**DOI:** 10.1042/BSR20171300

**Published:** 2018-03-16

**Authors:** Xiangjun Huang, Lamei Yuan, Hongbo Xu, Wen Zheng, Yanna Cao, Junhui Yi, Yi Guo, Zhijian Yang, Yu Li, Hao Deng

**Affiliations:** 1 Department of General Surgery, The First Affiliated Hospital of Hunan University of Chinese Medicine, Changsha, China; 2 Center for Experimental Medicine, The Third Xiangya Hospital, Central South University, Changsha, China; 3 Department of Neurology, The Third Xiangya Hospital, Central South University, Changsha, China; 4 Department of Ophthalmology, The Third Xiangya Hospital, Central South University, Changsha, China; 5 Department of Medical Information, Information Security and Big Data Research Institute, Central South University, Changsha, China

**Keywords:** ABCA4, exome sequencing, inherited retinal degeneration, mutation, retinitis pigmentosa

## Abstract

Retinitis pigmentosa (RP) is a group of hereditary, degenerative retinal disorders characterized by progressive retinal dysfunction, outer retina cell loss, and retinal tissue atrophy. It eventually leads to tunnel vision and legal or total blindness. Here, we aimed to reveal the causal gene and mutation contributing to the development of autosomal recessive RP (arRP) in a consanguineous family. A novel homozygous mutation, c.4845delT (p.K1616Rfs*46), in the ATP-binding cassette subfamily A member 4 gene (*ABCA4*) was identified. It may reduce ABCA4 protein activity, leading to progressive degeneration of both rod and cone photoreceptors. The study extends the arRP genotypic spectrum and confirms a genotype–phenotype relationship. The present study may also disclose some new clues for RP genetic causes and pathogenesis, as well as clinical and genetic diagnosis. The research findings may contribute to improvement in clinical care, therapy, genetic screening, and counseling.

## Introduction

Retinitis pigmentosa (RP, OMIM 268000) is a heterogeneous group of hereditary, degenerative retinal disorders with the estimated worldwide prevalence of 1/3000–1/7000. It affects approximately 0.1% of population in China [1,2]. Its features include night blindness, narrowed vision fields, gradually decreasing visual acuity, and fundus lesions eventually leading to tunnel vision and legal blindness [3,4]. It is frequently accompanied by cataracts, astigmatism, myopia, keratoconus, and hearing impairment except for those with Usher syndrome, which is characterized by RP and congenital deafness [5]. Pathologic features include progressive rod photoreceptor cell atrophy which leads to secondary cone death [6]. Histological characteristics are inner and outer retina disorganization with retinal ganglion cell death and vascular abnormalities, including perivascular cuffing, arteriolar attenuation, and decreased ocular blood flow [7]. RP is both clinically and genetically heterogeneous, inherited following Mendelian inheritance patterns. The most common RP inheritance pattern is autosomal recessive (50–60%), followed by autosomal dominant (30–40%), and X-linked trait (5–15%) [1,8,9]. Additionally, mitochondrial, *de novo*, and digenic mutations are reported in rare cases [10,11]. RP is thought to be caused by gene mutations which disrupt photoreceptor function or architecture [12]. The products of these genes participate in various molecular signal pathways, including, but not limited to, photoreceptor development, retinoid cycle, phototransduction, cilia, outer segment development, and protein transport [13].

Mutations in the ATP-binding cassette subfamily A member 4 gene (*ABCA4*, previously called *ABCR*, OMIM 601691) have been described as being responsible for a series of abnormalities, including cone-rod dystrophy 3 (CORD3, OMIM 604116), RP19 (OMIM 601718), age-related macular degeneration 2 (OMIM 153800), fundus flavimaculatus (FFM), Stargardt disease 1 (STGD1), and early-onset severe retinal dystrophy (OMIM 248200) [14–19]. Various *ABCA4* gene mutations ranging from point mutations to complex rearrangements have been identified, including missense, nonsense, splicing, insertion, deletion, and complex rearrangement mutations.

The present study is aimed at revealing the causative gene and mutation for the occurrence of autosomal recessive RP (arRP) in a consanguineous family with third-generation intermarriage. A novel homozygous mutation, c.4845delT (p.K1616Rfs*46), in the *ABCA4* gene was detected in the pedigree, with progressive degeneration of both rod and cone photoreceptors with arRP clinical features.

## Materials and methods

### Study participants and clinical evaluation

**Figure 1:**
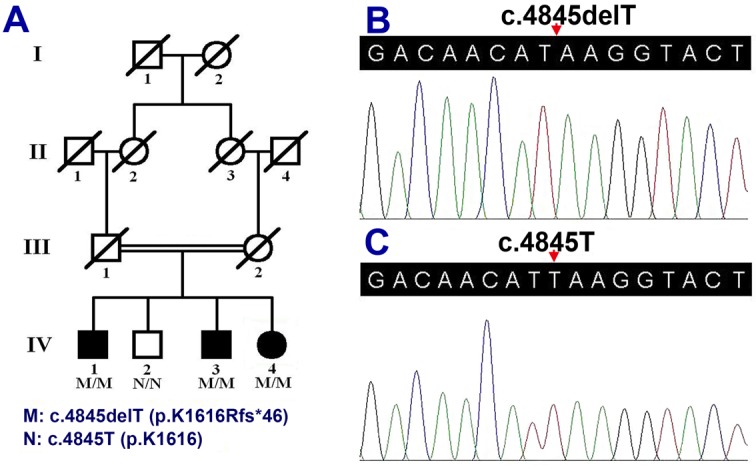
The mutation c.4845delT (p.K1616Rfs*46) in the *ABCA4* gene in a pedigree with RP (**A**) Pedigree of the family with arRP. Double lines indicate consanguineous unions. Square denotes male family member, circle represents female family member, slashed symbol indicates deceased family member, fully shaded symbol shows patient with RP, and open symbol presents RP-free member. (**B**) The *ABCA4* sequence with homozygous c.4845delT (p.K1616Rfs*46) mutation (IV:4). (**C**) The normal *ABCA4* sequence of RP-free member (IV:2).

Members of a four-generation Han Chinese pedigree with arRP, consisting of 12 individuals, were enrolled for genetic screening, at the Third Xiangya Hospital, Central South University, Changsha, China (Figure 1A). Peripheral venous blood of four available family members was sampled for the genetic study. All medical records of healthcare, routine physical, and fundus examinations were collected. Blood samples were also obtained from 100 independent ethnically matched normal controls (50 males and 50 females, age: 35.2 ± 4.3 years). Informed written consent was obtained from all participating subjects in the present study. All participants had ophthalmologic examinations performed. The examinations included E decimal charts, visual field evaluations, slit-lamp biomicroscopy, and fundus inspection. The arRP diagnosis was determined according to manifestations of progressive visual field constriction, nyctalopia, typical fundus findings, and similar symptoms in family members. The present study was approved by the Institutional Review Board of the Third Xiangya Hospital, Central South University, Changsha, China.

### Exome capture

Genomic DNA was separated from peripheral venous blood cells using standard DNA phenol-chloroform extraction procedures. Exome sequencing was carried out in the proband (IV:4) of the family by Novogene Bioinformatics Institute (Beijing, China). The genomic DNA was fragmented with a Covaris Ultrasonic Sample Processor (Covaris, MA, U.S.A.). A paired-end DNA library was constructed and the whole exome capture was performed with the SureSelect Human All Exon V6 Kit (Agilent Technologies Inc., Santa Clara, CA, U.S.A.). After quality assessment, the captured DNA library was sequenced on the Illumina HiSeq 2000 platform following the Illumina protocols (Illumina Inc., San Diego, CA, U.S.A.) [20].

### Variant analysis

After the base calling and quality assessment of sequencing data, alignment to human reference genome UCSC (GRCh37/hg19) was carried out for the effective sequencing data obtained using Burrows–Wheeler Aligner. SAMtools and Picard tools were applied to sort sequencing alignments and mark duplicate reads, respectively. Single nucleotide polymorphisms (SNPs) with an alignment rate of ≥95% and a read depth of ≥10× were rated as ‘high confidence’ [21–23]. Based on the variant information, the called SNPs and indels were separated into different functional categories using the Annotate Variation annotation. Generally, variants associated with monogenic disorders are rare in public variant databases. With specific settings, variants were filtered using datasets from the SNP database (dbSNP, build 142), 1000 Genomes Project (2014 September release), the National Heart, Lung, and Blood Institute Exome Sequencing Project 6500 (NHLBI ESP6500), and the Exome Aggregation Consortium (ExAC).

After removing common variants, retained variants were recognized as ‘novel’ variants. Only variants, including SNPs and indels, located in exonic regions or in canonical splicing sites, were deemed plausible candidates and prioritized for further analysis. *In silico* analyses, including Sorting Intolerant from Tolerant (SIFT), Polymorphism Phenotyping version 2 (PolyPhen-2), MutationTaster, and Combined Annotation Dependent Depletion (CADD), were employed to obtain functional prediction. A left-plausible, candidate-gene variant associated with vision disorders was then prioritized for confirmation in the validation stage [24–27]. Locus-specific candidate primers were designed using the Primer3 (version 4.0.0) online software. The oligonucleotides were synthesized by Sangon Biotech (Shanghai) Co., Ltd. Direct Sanger sequencing for mutation validation was carried out on an Applied Biosystems 3500 genetic analyzer [26]. The primers were synthesized as listed: 5′-CAGGAGGAGGGATGGAATTT-3′ and 5′-AAAACCGTCTTGTTTGTTTGTTT-3′.

## Results

### Clinical findings

**Figure 2:**
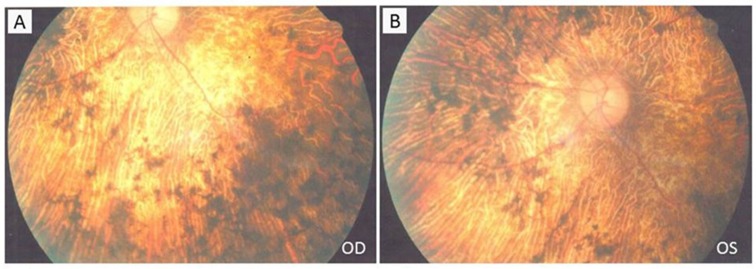
Fundus photographs of the patient (IV:1) with RP Fundus photographs of both eyes (A: right eye, B: left eye) present bone spicule-like pigmentation, pale fundus, optic nerve atrophy, retinal vessel attenuation, and retinal pigment epithelial degeneration.

Affected individuals (IV:1, IV:3, and IV:4, Figure 1A), including two males and one female, manifested similar clinical and funduscopic abnormalities including night blindness, decreased visual acuity, constricted vision field, waxy-pale discs, retinal vessel attenuation, and retinal degeneration. Patient IV:1 had night blindness and began having blurry vision at about the age of 15. This was followed by progressive visual field constriction before age 40. Patient IV:3 had progressive vision decrease beginning at 15, followed by tunnel vision. Patient IV:4 began having poor vision at night and blurred distance vision at 16 (Table 1). Fundus examinations showed bone spicule-like pigmentation, pale fundus, optic nerve atrophy, retinal vessel attenuation, retinal pigmented epithelium (RPE) degeneration, and macular involvement (Figure 2). Fundus examination of non-RP individual IV:2 showed no pigment migration, but did show unrelated right eye corneal conjunctivalization and left eye age-related cataracts, as determined by two independent ophthalmologists.

**Table 1:** Clinical features of individuals affected with RP

Individual	IV:1	IV:3	IV:4
Sex	M	M	F
Current age (years)	67	48	41
Age at onset (years)	15	15	16
VA (OD/OS)	LP/HM	LP/HM	HM/HM
Initial symptoms	Decreased vision, needs for more light	Decreased vision, needs for more light	Decreased vision, needs for more light
Ocular features	No abnormalities	Nystagmus, oculomotor apraxia	No abnormalities
Fundus features	A pale fundus, optic nerve atrophy, vessel attenuation, and retinal pigment epithelial degeneration	Bone spicule-like pigmentation, retinal vascular attenuation, and macular pigment alterations	Optic nerve atrophy, vessel attenuation, and retinal pigment epithelial degeneration

Abbreviations: F, female; HM, hand movement; LP, light perception; M, male; OD, right eye; OS, left eye; VA, visual acuity.

### Exome sequencing

Approximately 6.83 GB of raw data were generated by base calling. After the base calling and quality assessment, 45.24 million reads (100%) were generated from proband (IV:4) genomic DNA samples. There were 45.21 million reads (99.94%) aligned to the human reference assembly, and 4442.05 MB effective sequences were on the target region. The average sequencing depth on target region was 73.47 [20,26]. There were 60.22 million covered bases on the target region, meaning that the aligned bases covered 99.60% of the target region. The fraction of bases covered by the target sequence at 10× or greater was 98.70%. A total of 119268 SNPs and 14179 indels, including 21337 SNPs and 552 indels in exonic regions, and 2320 SNPs and 416 indels in splicing sites, were obtained. A variant filtration prioritization strategy, as described in recent studies, was used for variant analysis [26]. Common variants with a known frequency recorded in public databases were eliminated, which included dbSNP142, 1000 Genomes Project with a frequency of >0.01, the NHLBI ESP6500, and the ExAC, as well as synonymous variants. Non-synonymous variants for any likely pathogenicities were obtained from combining SIFT, PolyPhen-2, MutationTaster, and CADD analyses predictions. Using these filtering criteria, 507 possible deleterious SNPs and 312 indels were suspected as possible causative variants and were prioritized for further screening analysis. Except for a homozygous c.4845delT variant in the *ABCA4* gene (NM_000350.2), no other homozygous variants or compound heterozygous variants, which were in known disease-causing genes for vision and retinal degeneration disorders, were detected.

### Identification of causative mutation

Using Sanger sequencing, the novel homozygous variant, c.4845delT, in the *ABCA4* gene, was further verified in three patients (IV:1, IV:3, and IV:4, Figure 1B). It was recorded and public in Leiden Open Variation Database v.3.0 (http://www.lovd.nl/3.0/home) after the submission. The *ABCA4* gene variant was absent from an unaffected family member (IV:2, Figure 1C) and 100 unrelated controls. The computer-based prediction tool, MutationTaster, predicted that the *ABCA4* gene c.4845delT variant would lead to arginine substitution for lysine at codon 1616 resulting in a premature truncation at codon 1661 (p.K1616Rfs*46), and be disease causing.

## Discussion

RP is a class of inherited degenerative retinal diseases characterized by progressive retinal dysfunction, outer retina cell loss, and retinal tissue atrophy, eventually leading to tunnel vision and legal or total blindness [7,28]. Clinical features include adolescent-onset night blindness, limited visual fields, decreased visual acuity, and degenerative fundus change, followed by progressive peripheral vision loss, and culminating in adult blindness. RP can come in three forms: early-onset, late-onset, and non-penetrant [28,29].

In this consanguineous Han Chinese family with arRP, a homozygous variant, c.4845delT (p.K1616Rfs*46), in the *ABCA4* gene was identified. Severe phenotypes and early-onset ages were noticed in three affected family members: IV:1, IV:3, and IV:4. All showed typical RP clinical features, including adolescent-onset night blindness, followed by visual field loss, tunnel vision, and ultimately blindness. Disease severity is equal between male and female patients. These symptoms and the identified c.4845delT variant were absent from an unaffected sibling (IV:2). The variant was present in three patients, and absent from an unaffected family member, the 100 normal controls, the dbSNP142, the 1000 Genomes Project, NHLBI ESP6500, and ExAC. This suggests that it may be a pathogenic mutation. *In silico* analyses indicate that the mutation is probably deleterious. The *ABCA4* compound heterozygous mutations, an exonic deletion, and a heterozygous c.4845delT variant, cannot be fully excluded for the three affected siblings, due to the unavailable genotypes of the deceased parents and limitation of the detection methods applied in this study [30]. Genetic reasons, such as homozygosity, caused by uniparental disomy, should also be considered [31]. The homozygous *ABCA4* c.4845delT alteration was likely to be the responsible variant for arRP in this family due to the consanguinity of deceased parents.

The *ABCA4* gene, mapped to chromosome 1p22.1, contains 50 exons encoding a 2273-amino acid protein, expressed in retinal outer segments of rod and cone photoreceptors. The protein is located in the rod and cone outer segment disc membranes, and participates in the transport and clearance of all-*trans*-retinal, and other molecules passing through the disc membrane into the cytoplasm [32,33].

*ABCA4* gene mutations have been found to be responsible for five autosomal recessive retinal dystrophy phenotypes, including CORD3, RP19, FFM, STGD1, and early-onset severe retinal dystrophy [14–17]. The most severe phenotype, RP19 (arRP), has direct injures in both rod and cone photoreceptors [34]. At least 41 mutations, including 27 missense/nonsense mutations, 9 splicing mutations, 4 deletions, and a complex rearrangement, in homozygous or compound heterozygous state, have been reported to be responsible for arRP, by the Human Gene Mutation Database and in the published literature [35–38]. Several *ABCA4* heterozygous mutations have been reported in a few RP cases. A second causative mutation, such as deep intronic disease-associated variants, gross deletion, or large rearrangement, might not be detected due to limited genotyping methods. Seemingly benign or common variants, as well as hypomorphic variants, should also be considered [39–42]. *ABCA4*-related disease may result from the all-*trans*-retinal accumulation in the photoreceptor discs owing to reduced activity of ABCA4 protein, which ultimately results in RPE cell death and secondary loss of photoreceptors [43,44].

*Abca4*^−/−^ and *Abca4*^+/−^ mice showed delayed dark-adaptation and RPE lipofuscin accumulation, with no photoreceptor degeneration [45,46]. Double-knockout mice, which lacked both *Abca4* and retinol dehydrogenase 8, displayed all-*trans*-retinal accumulation and early severe RPE/photoreceptor dystrophy. The retinopathy was exacerbated by light [47].

In conclusion, a novel homozygous mutation c.4845delT (p.K1616Rfs*46) in the *ABCA4* gene was detected in a Han Chinese family with arRP. These findings extend both the arRP genotypic and the *ABCA4* gene mutation spectrum. It suggests that exome sequencing is a valid and cost-effective way of identifying gene mutations that may be responsible for genetically, and clinically, heterogeneous disorders. The study may disclose some new clues for RP genetic causes and pathogenesis, as well as clinical and genetic diagnosis. This may contribute to improvement in clinical care, therapy, genetic screening, and counseling, and assist in developing targetted gene therapeutic strategies for RP.

## Abbreviations

ABCA4: ATP-binding cassette subfamily A member 4
arRP: autosomal recessive retinitis pigmentosa
CADD: Combined Annotation Dependent Depletion
CORD3: cone-rod dystrophy 3
dbSNP: single nucleotide polymorphism database
ExAC: Exome Aggregation Consortium
FFM: fundus flavimaculatus
NHLBI ESP6500: National Heart, Lung, and Blood Institute Exome Sequencing Project 6500
PolyPhen-2: Polymorphism Phenotyping version 2
RP: retinitis pigmentosa
RPE: retinal pigmented epithelium
SIFT: Sorting Intolerant from Tolerant
SNP: single nucleotide polymorphism
STGD1: Stargardt disease 1
